# Improving Drug Safety in Pediatric and Young Adult Patients with Hemato-Oncological Diseases: A Prospective Study of Active Pharmacovigilance

**DOI:** 10.3390/ph17010106

**Published:** 2024-01-12

**Authors:** Anna Parzianello, Giulia Fornasier, Valentina Kiren, Federico Pigato, Sabrina Orzetti, Giulia Zamagni, Anna Arbo, Paolo Baldo, Paola Rossi, Marco Rabusin, Maurizio Mascarin, Marta Paulina Trojniak

**Affiliations:** 1 Department of Medicine, Surgery and Health Sciences, Postgraduate School of Clinical Pharmacology and Toxicology, University of Trieste, 34127 Trieste, Italy; anna.parzianello@studenti.units.it; 2 Regional Agency for Health Coordination of Friuli Venezia Giulia (ARCS), 33100 Udine, Italy; giulia.fornasier@arcs.sanita.fvg.it; 3 Pediatric Hemato-Oncology Unit, Institute for Maternal and Child Health—IRCCS Burlo Garofolo, 34137 Trieste, Italy; valentina.kiren@burlo.trieste.it (V.K.); marco.rabusin@burlo.trieste.it (M.R.); 4 Pharmacy Unit, Institute for Maternal and Child Health—IRCCS Burlo Garofolo, 34137 Trieste, Italy; federico.pigato@burlo.trieste.it (F.P.); anna.arbo@burlo.trieste.it (A.A.); 5 Pharmacy Unit, IRCCS CRO National Cancer Institute, 33081 Aviano, Italy; sabrina.orzetti@cro.it (S.O.); pbaldo@cro.it (P.B.); 6 Clinical Epidemiology and Public Health Research Unit, Institute for Maternal and Child Health—IRCCS Burlo Garofolo, 34137 Trieste, Italy; giulia.zamagni@burlo.trieste.it; 7 Central Directorate for Health, Social Policies and Disability—Friuli Venezia Giulia Region, Regional Center for Pharmacovigilance, 34121 Trieste, Italy; paola.rossi@regione.fvg.it; 8 Integrated Oncology for Adolescents and Young Adults and Pediatric Radiotherapy Unit, IRCCS CRO National Cancer Institute, 33081 Aviano, Italy; mascarin@cro.it

**Keywords:** pharmacovigilance, adverse drug reaction, hemato-oncological disease, pediatric population, safety, multidisciplinary team

## Abstract

The acquisition of relevant pediatric clinical safety data is essential to ensure tolerable drug therapies. Comparing the real number of Adverse Drug Reaction (ADR) reports in clinical practice with the literature, the idea of ADR underreporting emerges. An active pharmacovigilance observational prospective study was conducted to assess the safety of oncology pharmacological prescriptions in patients aged 0–24 years at Institute for Maternal and Child Health IRCCS Burlo Garofolo in Trieste and IRCCS CRO National Cancer Institute in Aviano (Italy) between January 2021 and October 2023. Prescriptions and ADRs were evaluated by a multidisciplinary team. A total of 1218 prescriptions for 38 patients were analyzed, and 190 ADRs of grade 3–5 were collected. As compared to historical data, we registered a significant increase (*p* < 0.001) in the number of ADRs. The risk of ADR was 3.4 times higher in the case of off-label prescriptions compared to on-label ones (OR 3.4; [1.47; 7.89]; *p*-value = 0.004). The risks of error and near-miss were reported for 6.3% and 18.2% of total prescriptions, respectively. Of the total of 133 interactions, 47 (35.3%) resulted in ADRs. This study shows the importance of pro-active pharmacovigilance to efficiently highlight ADRs, and the fundamental role of multidisciplinary teams (oncologist, pharmacist, pharmacologist, pediatrician, nurse) in improving patients’ safety during therapy.

## 1. Introduction

Current oncology treatment protocols for pediatric and young adult patients with hemato-oncological diseases, while allowing high cure rates, are extremely invasive and expose the oncological young patient to the risk of toxicity and unexpected adverse reactions that, rarely, can prove fatal. Fundamental to the prevention of these risks is both the close collaboration between health care professionals, ensuring careful monitoring of the patient’s clinical data combined with the pharmacological characteristics of drugs, and the involvement of patients and families in the care process, particularly in home drug management, through adequate information that enables them to be made aware of the proper and correct use of the drugs.

Adverse Drug Reaction (ADR) reporting is the most important source of information that allows professionals to gain a thorough understanding of drug use, with the goal of making them safer for patients [1]. Collecting an adequate number of ADR reports is the best strategy to identify an alarm signal in a timely manner. In this regard, the Italian Ministry of Health’s Recommendation n. 17 provides tools to ensure and increase drug safety, focusing primarily on recommendations for drug reconciliation; the prevention of errors in antineoplastic drug therapy; the prevention of errors in “look-alike/sound-alike” drug therapy; and the prevention of death, coma or serious injury resulting from errors in drug therapy [2].

Based on the literature, active surveillance in this context seems to be an excellent strategy to improve therapeutic appropriateness. In fact, the active monitoring of drug prescriptions ensures that the patient takes the most appropriate drug in the correct dose, route of administration and duration of treatment, thus limiting therapeutic errors [3,4,5,6,7,8].

Focusing on active monitoring studies in the adult and pediatric oncology literature, serious ADRs are estimated to occur in about 20–30% of patient prescriptions [9,10], while therapeutic errors are in about 20% [11,12]. In this view, the active role of clinical pharmacists within a multidisciplinary oncology team could be a valuable support to the governance of oncological drug therapy, thereby improving safety and clinical outcomes in this vulnerable patient population.

Because pediatric patients represent a vulnerable population, it is extremely necessary that only drugs tested for a specific setting are used in a given pediatric population. Thus, the risks of side effects that may occur during a pharmacological therapy may be potentially known, prevented and eventually managed. In this view, the importance of prescriptive appropriateness should be emphasized, and as far as possible, drugs should be used according to the SmPC.

However, given the very peculiarities of the pediatric population, it is plausible that in the absence of valid therapeutic alternatives physicians should prescribe off-label drugs: drugs approved for other indications and/or other age groups. As a consequence of this inappropriate drug use, there may be a greater likelihood of unexpected side effects that may be difficult to prevent and manage, and that may hinder the effectiveness of the pharmacological treatment. In any case, off-label drugs should be prescribed based on reliable results published in the scientific literature, as specified by Italian L.94/98 [13].

Actually, greater therapeutic safety is certainly provided by the use of drugs under Italian L 648/96 [14]. Law 648/96 accounts for the evidence-based use of drugs in specific unauthorized settings, thus representing in many cases a valid therapeutic alternative prior to an off-label drug.

With regard to drug safety and efficacy, drugs’ interactions should also be considered. All of the drugs the patient is taking should be considered in the drug interaction analysis, both the prescribed drugs and those self-administered by the patient. Hence, for example, the importance of constant communication and collaboration between patients/their families and the physician, providing him with all the available information.

In this view, IRCCS for Maternal and Child Health Burlo Garofolo in Trieste (Italy) and IRCCS CRO National Cancer Institute in Aviano (Italy) conducted a prospective study to assess whether the introduction of the active monitoring of the multidisciplinary team on pediatric and young adult oncology prescriptions would increase the number of ADR reports and therapeutic safety. While the first aim of this study was to quantify the number of grade 3–5 ADR reports compared to the total prescriptions analyzed, and to compare these data with historical ones, the secondary aims were to quantify ADRs due to off-label drug use, therapeutic errors and interactions based on the total prescriptions.

## 2. Results

The primary and secondary outcomes of this study were achieved. The study enrolled a total of 38 patients with hemato-oncological diseases, of whom 24 (63.2%) were male. The mean age was 9.8 (SD 5.8) years, and, considering primary and secondary outcomes, there were no statistically significant differences between the age groups. Thirty five (92.1%) patients were inpatients, two (5.3%) patients were enrolled in day hospital settings and one (2.6%) patient was an outpatient. The most frequent diagnosis among the enrolled patients was acute lymphoid leukemia. All diagnoses of enrolled patients and their frequencies are given in Table 1. Eight (21.1%) patients had comorbidities. One patient was lost during the follow-up because he was in the terminal phase and the oncologist, in agreement with the patient, did not administer chemotherapy, but only supportive therapy.

With regard to primary outcomes, a total of 1218 prescriptions of oncology drugs to enrolled patients were collected and analyzed during the study period, of which 996 prescriptions coming from IRCCS Burlo and 222 from IRCCS CRO. A total of 190 (15.6%) of the total prescriptions resulted in at least one ADR, and these occurred in 32 (86.5%) enrolled patients. Of the total ADRs collected, 79 (60.8%) were reported as serious; compared to historical data from 2017 to 2018, the increase in the number of ADRs from 35 (2.8%) to 190 (15.6%) was statistically significant (*p* < 0.001).

With regard to the secondary outcomes, 674 (55.3%) drugs were prescribed according to the SmPC, and 544 (44.7%) were beyond the authorized indications (L. 648/96, L.648/96 consolidate use and off-label L. 94/98); a total of 24 (2.0%) prescriptions were off-label, and nearly 20% of patients experienced at least one ADR due to off-label drugs. The risk of ADR was 3.4 times higher in the case of off-label prescriptions compared to on-label ones (OR 3.4; [1.47; 7.89]; *p*-value = 0.004). The risk of error and near-miss were reported for 77 (6.3%) and 222 (18.2%) of total prescriptions, respectively. Thirty one (83.8%) patients experienced at least one drug–drug interaction. Of the total 133 interactions (65 pharmacokinetics and 68 pharmacodynamics), 47(35.3%) resulted in ADRs, and this occurred at least once in 24 (77.4%) patients with interactions.

At IRCCS Burlo, out of a total of 996 oncologic prescriptions, at least one ADR occurred in 116 (11.6%) prescriptions. Considering the historical data of years 2017–2018, out of a total of 1260 oncologic prescriptions, at least one ADR occurred in 35 (2.8%) prescriptions. Thus, the proportion of ADRs to total oncologic prescriptions increased from 2.8% to 11.6%. The increase was found to be statistically significant (*p*-value < 0.001). At IRCCS CRO, out of a total of 222 total oncologic prescriptions, at least one ADR occurred in 74 (33.3%) prescriptions. Considering the historical data of years 2017–2018, out of a total of 831 oncologic prescriptions, no ADRs occurred. Thus, the proportion of ADRs to total oncologic prescriptions increased from 0 to 33.3%. Again, the increase was found to be statistically significant (*p*-value < 0.001). All these data are presented in Table 2.

Out of a total of 1218 oncologic prescriptions, at least one ADR occurred in 190 (15.6%) prescriptions. Considering these 190 oncology drug prescriptions, more than half were for four drugs, such as methotrexate, doxorubicin, ifosfamide and vincristine. Table 3 shows the frequencies of drug prescriptions with at least one associated ADR, and, for each, the most frequent undesirable effects.

Out of a total of 37 patients who took oncology drugs (1 patient was lost during follow up), at least one ADR occurred in 32 (86.5%) patients. Out of a total of 1218 prescriptions, 674 (55.3%) drugs were prescribed according to the SmPC, and 544 (44.7%) were beyond authorized indications. These were off-label uses with good clinical evidence, and were already authorized and reimbursed in Italy under Law 648/1996. More in detail, 342 (28.1%) drugs were prescribed according to Law 648/96, and 178 (14.6%) drugs prescribed according to Law 648/96 on consolidate use. Finally, 24 (2.0%) drugs were prescribed off-label. Of these 24 off-label prescriptions, 13 (54.2%) were off-label due to age, and 11 (45.8%) were due to indication. The risk of ADR was 3.4 times higher in the case of off-label prescriptions compared to on-label ones ([1.47; 7.89]; *p*-value = 0.004). The risk of ADR was 1.5 times higher in the case of prescriptions outside authorized indications (OR 1.58; [1.15; 2.15]; *p*-value = 0.004) compared to SmPC use. The administered oncology drugs that resulted in one or more ADR due to on-label and off-label prescriptions are shown in Table 4.

Considering the total of 32 patients with at least one ADR, 6 experienced at least one ADR due to off-label oncology drugs, 26 due to on-label oncology drugs while 3 did due to on-label and off-label oncology drugs (considering that patients may have had multiple ADRs due to off-label and on-label prescriptions). Although no association was found (*p*-value = 0.561) between taking at least one off-label drug and having at least one ADR, especially because the sample was too small to achieve statistical significance, nearly 20% (19.4%) of patients who reported at least one ADR were also taking at least one off-label drug.

Despite a total of 190 prescriptions with at least one ADR, out of a total of 1218 prescriptions, a total of 130 ADRs were analyzed during the study period. Note that multiple drug prescriptions may be involved in an ADR and be assigned as suspected drugs. In fact, out of a total of 130 ADRs analyzed, 60 ADRs (46.2%) involved only one drug, 53 (40.7%) ADRs involved two drugs, while 17 (13.1%) ADRs involved three drugs. Considering the total of 32 patients with at least one ADR, in 10 (31.2%) patients the maximum number of drugs involved in ADR was three and in 18 (56.3%) patients it was two, while only in 4 (12.5%) patients there was only one drug involved per ADR. Table 5 shows the frequencies of collected ADRs classified according to SOC (System Organ Classes) and the total number of patients who manifested an ADR at least once. More than half of the ADRs (76 (58.5%) ADRs) involved the blood and lymphatic systems and the gastrointestinal tract. Of the 56 ADRs in 25 patients that affected the blood and lymphatic system, 28 (50%) consisted of neutropenic fever, which occurred at least once in 16 (64%) patients. Among the 20 ADRs in 15 patients that affected the gastrointestinal tract, the most frequent symptom was vomiting (7 (35%) ADRs), and it occurred at least once in 7 (46.7%) patients. Descriptions of these 76 ADRs with their frequencies and total number of patients in whom they occurred at least once are also given in Table 5.

Out of a total of 130 analyzed ADRs, 79 (60.8%) ADRs were reported as serious, of which 52 (65.8%) ADRs resulted in hospitalization or prolonged hospitalization. At least one serious ADR occurred in 27 (84.4%) patients. The most frequent outcome of the 130 total ADRs was complete resolution, which occurred in 81 (62.3%) ADRs. In 29 (90.6%) patients, complete resolution occurred at least once. Table 6 shows both the ADR outcome frequencies on the total of ADRs collected and the number of patients in whom they happened at least once.

Out of a total of 130 ADRs analyzed, only 1 (0.8%) ADR was due to overdose, although it was reported as not serious with a complete resolution where mercaptopurine was involved.

Considering the causal link between suspected drugs and ADRs evaluated with the Naranjo algorithm, it was found to be probable for most of the 130 analyzed ADRs (103 (79.2%) ADRs), it was highly probable for 16 (12.3%) ADRs and it was possible for 11 (8.5%).

Considering the risk of therapy for the total 1218 prescriptions, no risk of error was reported for 919 (75.5%) prescriptions, and it involved 32 (83.8%) patients. In contrast, a risk of error was reported for 77 (6.3%) prescriptions and happened at least once in 20 (54.1%) patients. Among these 77 prescriptions, in only 2 (2.6%) the risk of error was due to the incorrect drug administration. Both prescriptions to two different patients involved mercaptopurine. In addition, a near-miss was reported for 222 (18.2%) prescriptions and happened at least once in 6 (16.2%) patients.

During the study period, a total of 295 ancillary therapies for 37 patients were collected. All the patients enrolled in the study took at least one ancillary therapy. Actually, they took an average of eight (IQR 5–10) ancillary therapies each. Among the 295 ancillary therapies, the most frequent drug cluster was antibiotics. In fact, 97 (32.9%) ancillary drugs were antibiotics, which were taken at least once by 34 (91.9%) patients.

Out of a total of 37 patients with oncology therapies, 133 interactions (65 pharmacokinetics, and 68 pharmacodynamics) were found in 31 (83.8%) patients, who experienced at least one interaction each. Actually, the median number of interactions per patient was four (IQR 1–6). The 266 therapies resulted in 133 interactions and involved 55 drugs, of which 16 (29.1%) were oncology drugs. The drugs involved in these 266 therapies and their frequencies are presented in Table 7.

Out of a total of 133 interactions, the most frequent involved methotrexate with sulfamethoxazole/trimethoprim (16 (12.0%)), methotrexate with amoxicillin/clavulanate (11 (8.3%)), and methotrexate with mercaptopurine (9 (6.8%)), while 10 (7.5%) interactions were between mercaptopurine and sulfamethoxazole/trimethoprim.

Out of a total of 133 interactions, 47 (35.3%) resulted in ADRs, and this happened at least once in 24 (77.4%) patients. A statistically significant association (*p*-value < 0.001) was found between the interactions resulting in ADRs and the type of interaction. Out of a total of these 47 interactions that resulted in ADRs, 34 (72.3%) were due to pharmacokinetics and happened at least once in 18 (75.0%) patients, and 13 (27.7%) interactions were due to pharmacodynamics and happened at least once in 11 (45.8%) patients. Considering the risk rating for the 47 interactions resulting in ADRs, for 2 (4.3%) interactions the drugs’ combination should have been avoided, and this happened at least in 2 (8.3%) patients. For 33 (70.2%) interactions, it was sufficient to monitor therapy, while for 12 (25.5%) interactions a change in therapy should have been considered, and this happened at least once in 10 (41.7%) patients. All these data are presented in Table 8.

## 3. Discussion

The primary endpoint of this study was to evaluate the prescriptive and safety profiles of drug treatment in children and young adults with hemato-oncologic diseases, based on the number of ADRs that occurred out of total oncology prescriptions. In addition, we focused on the characterization of ADRs, drug prescriptions and drug–drug interactions.

In this study, a statistically significant (*p*-value < 0.001) increase in the proportion of ADRs to total oncological prescriptions was reported for both Institute. At IRCCS Burlo, it increased from 2.8% to 11.6%, while at IRCCS CRO it increased from 0 to 33.3%. The difference in clinical practice between the two considered periods lies in the adoption of an active pharmacovigilance process by the multidisciplinary teams. Considering historical data, the consistent idea of ADR underreporting in both institutes emerged, especially at IRCCS CRO. However, this study proves that even just few improvement actions in clinical practice, as the pro-active collaboration between oncologists and clinical pharmacists, may result in the increased identification and reporting of ADRs.

ADRs are the main pharmacovigilance issue in the oncology setting, as oncological drug toxicity is considered common and an expected consequence of these therapies. This oncological drug toxicity also emerged consistently in our study. As this is a very frequent phenomenon, the real need is to develop a strategy to minimize the occurrence of serious ADRs, which may harm the patient’s life or may reduce the effectiveness of drug therapy, mainly by the monitoring of therapeutic appropriateness and drug interactions. As for less severe ADRs, these can be managed primarily with appropriate patient/caregiver education. Confirming the high frequency of ADR occurrence, in this study at least one ADR occurred in 86.5% of patients (32 patients out of 37). Hence, the need to make pediatric patients and their families aware of the importance of pharmacovigilance actions to increase the safety and effectiveness of pharmacological oncology therapies. In line with the literature, the most frequent ADRs were neutropenic fever and vomiting. Considering the ADRs collected, multiple drug prescriptions were involved in the same ADR and were assigned as suspected drugs. Actually, less than half of analyzed ADRs (46.2%, 60 out of 130) involved only one drug. These results alone show it that the prescribing doctor must consider all drugs taken by the patient before making a new prescription. The problem of polypharmacotherapy cannot be underestimated in these patients, considering how they take at least one oncology drug, but, most of the time, more than one, because of the specific oncology protocols, in addition to supportive therapy and therapies for any other patient-specific clinical conditions. Indeed, as also confirmed by the results of this study, ADRs to oncology drugs are very frequent and may require drugs for their treatment and/or prevention. Especially because supportive therapies may vary during oncological treatment, in clinical practice drug interactions should be checked whenever necessary. In this regard, it should become a clinical practice to analyze drug interactions, also considering the foods or supplements taken by the patient. In our study, the median number of interactions occurring per patient was four (IQR 1–6), and most of the patients who received oncological therapies (83.8%, 31 out of 37 patients) experienced at least one interaction each. In 24 patients, at least one drugs interaction resulted in ADRs. Thus, in clinical practice, all possible interactions, alterative drugs and changes in therapy should be evaluated, and this may be fully investigated thanks to the close collaboration within multidisciplinary teams [6,9,15]. Moreover, when considering relapsed patients, there is also the possibility that strict oncology protocols are not followed, but, with the goal of slowing disease progression and increasing quality of life, physicians resort to prescriptions outside indications. A very close collaboration among health care professionals is needed to ensure the safety of these pharmacological treatments. Despite the small sample size, two ADRs due to the incorrect administration of oral oncology drugs at home were collected in this study, including one due to an overdose. This result suggests that patient/caregiver education can also make a difference in terms of safe drugs use.

In this study, the risk of ADR was found to be 1.5 times higher in case of prescriptions outside authorized indications (OR 1.58; [1.15; 2.15]; *p*-value = 0.004) compared to SmPC use, and, more in detail, the risk of ADRs was found to be 3.4 times higher in case of off-label prescriptions compared to on-label ones ([1.47; 7.89]; *p*-value = 0.004). Half of therapies (55.3%, 674 out of 1218) were prescribed according to the SmPC, while the other half were beyond authorized indications. Although this type of prescription may result in a major likelihood of ADR occurrence, as the literature and our results confirm, it must be considered here in the population involved. As already known, the pediatric population represent a vulnerable population, often characterized by the lack of specific authorized therapies. In this way, the use of drugs beyond authorized indications represents a viable alternative in the pediatric setting. However, although the most frequent outcome also that occurred in our study was a complete resolution of ADRs, at least considering short follow-ups, once again patients and their families must be adequately informed about the risks involved in this type of prescription [7,16].

In hospital settings, multidisciplinary teams can adopt many strategies to prevent the side effects of oncological therapies. Certainly, collaboration with the pharmacist, who at the time of the drug prescription checks for prescriptive appropriateness, but who also should promptly assess the reported side effects, is essential. Furthermore, active pharmacovigilance is crucial to ensure medication safety and to decrease the phenomenon of under-reporting. In addition, the pharmacist can monitor potential interactions, and this should be a valuable support for the oncologist [15,17,18,19]. Moreover, to reach correct the blood concentrations of oncological drugs, the oncologist should make use of Therapeutic Drug Monitoring (TDM). In this way, the oncologist could obtain information of the patient-specific pharmacokinetics of the drug and use this data to adjust drug therapy in the best possible way, if necessary [20]. More in detail, the task of oncologists and pediatricians lies in describing as accurately as possible the patient’s clinical condition and all the therapies he or she is taking to the pharmacist and, if present, to the pharmacologist. In fact, they rely on the indications provided to them to conduct the prescriptive appropriateness analysis, to detect possible drug interactions or to hypothesize and thus prevent possible adverse reactions, to suggest dosage changes or possible drug monitoring to the clinician. All this should be carried out to ensure the safest and most effective drug therapy for the patient.

Considering patient/caregiver education and empowerment, one of the useful tools for obtaining adequate information and improving safety in the use of oral oncology drugs at home was the creation and distribution of booklets, regarding oral methotrexate, mercaptopurine and thioguanine, to the patients and their families upon discharge from the hospital. The health care professionals such as oncologists, pharmacists, pediatricians and nurses working closely with patients and their families play an important role in patient education and empowerment with the support of booklets, apps, etc.

The strength of these tools is surely the easy and immediate access to the available information, described in age-appropriate language; in fact, each booklet has a double editorial team, one aimed at parents and children, which also includes a comic for children, and one aimed at teenagers. In addition to the information on the correct administration of the oncological drugs, these booklets contain information on how to recognize, treat, and report adverse events, even the simplest and therefore underestimated ones, as well as information about possible interactions between the oncological drug and another drug the patient may be taking, and about interactions with any foods, including commonly consumed foods. Another strength of these tools is the ability for the patient/familiars to take notes on toxicity and safety issues encountered while using the drug, or to track treatment compliance. This way of proceeding may facilitate communication to the oncologist about the progress of oral drug treatment, thus assuring him comprehensive information on the safety of drug use. The next step to make this adequate information practical and relevant to everyday life is to convert the information printed on the booklets into a digital version through the development of an ad hoc application for mobile devices. This way of proceeding can certainly be useful in involving the patient themselves in the care process, also ensuring a more direct dialogue and communication with the clinicians. Thus, the innovation of social and health care processes may be also applied in the context of active pharmacovigilance [21,22].

In Italy, patients, citizens and caregivers, as well as healthcare professionals, can report adverse reactions to drugs and/or vaccines directly on the Italian regulatory agency (AIFA) website. Moreover, patients and their family members are instructed to report any symptoms and signs of concern to the physicians. If there is urgency, they directly notify the physicians; otherwise, they note them in the diary. To note, booklets support patients and caregivers especially in this process.

The strength of this study is that active monitoring was carried out on pediatric patients who are generally not taken into consideration in the approval studies for drugs’ authorization. In, addition, the involvement of a multidisciplinary team in the active pharmacovigilance process made it possible to combine patient clinical data with pharmacological data, resulting in a comprehensive view of the therapeutic scenario. Therefore, pro-active pharmacovigilance studies like this allow us to detect real adverse reactions and report them, avoiding under-reporting. Moreover, the safety of pharmacological oncology therapies collected in this study was evaluated in its entirety. In fact, the quali-quantitative analysis of ADRs was accompanied by the quali-quantitative analysis of both interactions and ancillary therapies, as well as the assessment of prescriptive appropriateness. Finally, an additional strength of this study lies in its prospective observational nature, which allowed the multidisciplinary team to follow patients from enrollment until the end of study period.

The main limitation of this study is the sample size. In fact, it is too small to generalize the results to the entire pediatric population, although they are in line with the literature. Further, larger studies are certainly needed to understand how the active pharmacovigilance process can be better improve in this oncological setting.

## 4. Materials and Methods

This is a prospective pharmacovigilance study conducted at IRCCS for Maternal and Child Health Burlo Garofolo in Trieste and IRCCS CRO National Cancer Institute in Aviano in the Friuli Venezia-Giulia region (Italy) form January 2021 to October 2023.

### 4.1. Study Population

The statistical unit was the pharmaceutical prescription to oncological patients aged < 25 years afferent to the Pediatric Oncology Department at IRCCS Burlo and to the Youth Area at IRCCS CRO in inpatient, day hospital and outpatient settings during the study period. Pharmaceutical prescriptions for the treatment of solid and hematological tumors were to be for cytotoxic drugs, targeted therapy and immunotherapy.

Written informed consent both for participation in the study and for the processing of personal data was signed by adult patients or by parents/legal representatives for minors.

### 4.2. Historical Data

Historical data revealed that the number of ADR reports entered into the National Pharmacovigilance Network (NPVN), connected with EudraVigilance Post-marketing Module (EVPM), by the Pediatric Oncology Unit of IRCCS Burlo in the years 2017 and 2018 were 13 grade 3–5 ADRs out of 564 oncology prescriptions (2.3%) and 22 grade 3–5 ADRs out of 696 oncology prescriptions (3.2%), respectively. For the Youth Area of the IRCCS CRO there were no grade 3–5 ADR reports out of the total of 444 prescriptions in 2017 (0%) and 387 in 2018 (0%).

Considering an average of 6 prescriptions of therapy cycles per patient, the percentage of patients with ADRs was 2.8% at IRCCS Burlo and 0% at IRCCS CRO.

### 4.3. Outcomes

The first aim of this study was to quantify the number of grade 3–5 ADR reports compared to the total prescriptions analyzed, which would not have been reported without active intervention, in the pediatric and young adult oncology field during the active monitoring period in both institutes, and to compare these data with historical ones (i.e., ADR reports entered in NPVN in 2017 and 2018 in both institutes). ADR grades are standardized using the Common Terminology Criteria for Adverse Events (CTCAE) v4.3.

The secondary aims were to quantify ADRs due to off-label drug use, therapeutic errors and interactions based on the total prescriptions in the pediatric and young adult oncology setting during the same study period.

### 4.4. Sample Size

The hypothesis of the study was to detect an increase in grade 3–5 ADR reports during the study period in both institutes compared to the total number of prescriptions collected. This data will be compared to the proportion of grade 3–5 ADR reports entered into NPVN of the total prescriptions of the years 2017 and 2018 in both institutes. Multiple prescriptions could be considered for the same patient at different times.

Considering IRCCS Burlo, to test the hypothesis of observing a difference in the proportion of ADRs of at least 3 percentage points (expected 6% compared to 3% observed in the 2017–2018 period), set an alpha significance level of 5% and a statistical power of 80%; the sample size should consist of 317 drug prescriptions (exact 2-tailed test for a proportion).

Considering IRCCS CRO, to test the hypothesis of observing a difference in the proportion of ADRs of at least 3 percentage points (expected 3.5% compared to the 0.5% conventionally set for the 2017–2018 period), assuming an alpha significance level of 5% and a statistical power of 80%, the sample size should consist of 91 drug prescriptions (exact 2-tailed test for a proportion).

### 4.5. Study Conduction

The clinical pharmacist involved in the study prospectively followed patients who were prescribed oncological drugs in collaboration with oncologists. The active role of the clinical pharmacist was to monitor each new prescription of oncological drug to enrolled patients. Specifically, monitoring activities consisted of checking the correctness of drug use and the appropriateness of prescription, assessing drug and therapy safety by detecting any grade 3–5 ADRs that occurred during the course of treatment, and detecting any errors, possible errors (near misses) or possible clinically significant drug–drug, drug–pathology and drug–food interactions through the consultation of the Lexicomp database, thus proposing to the prescribing oncologist the possible actions to be taken.

The pharmacological therapies are characterized by the concomitant administration of multiple oncology drugs and ancillary therapies. In the event of the onset of an ADR, the oncologist should assign the suspected drugs and the concomitant ones, as required by current legislation. In this view, data on concomitant and ancillary therapies were also collected and analyzed by the clinical pharmacist during the study period to assess the safety and appropriateness of concomitant administration with oncological drugs.

During the study period, the clinical pharmacist provided support for healthcare professionals to report grade 3–5 ADRs.

### 4.6. Data Collection

Data on drug prescriptions collected during the study period and the clinical data of patients to whom they refer were obtained by the clinical pharmacist from the consultation of medical records, referrals, discharge letters, nominal drug prescriptions and computerized nominal prescriptions sent to the Antiblastic Drugs Unit.

The collected data were entered in a standardized manner in the electronic case report form into a specific database (REDCap, version 13.1.33). Data collected were anonymized with an alphanumeric code identifying the patient to whom the prescription refers.

### 4.7. Statistical Analysis

Categorical variables were reported as counts and percentages, while continuous variables as mean and standard deviation or median and interquartile ranges, on the basis of the result of the normality test previously performed (Shapiro–Wilk). Differences between groups were evaluated using the Chi-square test (or Fisher’s exact test, when appropriate) in case of categorical variables, and the Student’s t test or the nonparametric analogue (i.e., Wilcoxon-Mann–Whitney) for continuous variables. A univariate logistic regression model was used to evaluate if the off-label assumption was a risk factor for the occurrence of ADR(s). Statistical significance was set at 0.05.

All the analyses were performed using the statistical software Stata (version 18).

### 4.8. Ethical Aspects

Only information related to prescriptions collected during the study period and clinical data of patients who have signed a valid consent form to the processing of personal and sensitive data for clinical research, epidemiology and training purposes were evaluated (regional consent form verified on the “GE.CO” system”).

The study was conducted in accordance with GCP, the ethical principles deriving from the Declaration of Helsinki and current legislation on observational studies. The study began after a favorable opinion was expressed by the Ethics Committee.

## 5. Conclusions

This study assessed the prescriptive and safety profiles of pharmacological treatment for children and young adults with hemato-oncology diseases, and showed the importance of pro-active pharmacovigilance in order to efficiently highlight ADRs. It also confirmed the increased risk of ADRs in the case of prescriptions outside the authorized indications. Moreover, the fundamental role of a multidisciplinary team in supporting patients during therapy emerged. In this context, the clinical pharmacist’s support for healthcare professionals is fundamental to decrease the phenomenon of under-reporting.

In the end, once the pro-active pharmacovigilance process in hospital settings has been consolidated, the next step to ensure the greater safety of pharmacological therapy will be the improvement of patient/caregiver education and empowerment on pharmacovigilance.

## Figures and Tables

**Table 1:** Diagnoses of enrolled patients.

Diagnosis	Patients (%) N = 38
Acute lymphoid leukemia	18 (47.4)
Edwing’s sarcoma	4 (10.5)
Chronic lymphoid leukemia	2 (5.3)
Hodgkin’s disease	2 (5.3)
Pleural synovial sarcoma	2 (5.3)
Other	10 (26.3)

**Table 2:** Proportion of ADRs to the total oncologic prescriptions for the years 2017–2018 and 2021–2023 at IRCCS Burlo and IRCCS CRO.

	2017–2018	2021–2023	*p*-Value
IRCCS Burlo			
Total prescriptions	N = 1260	N = 996	
ADR			<0.001
Yes (%)	35 (2.8)	116 (11.6)	
No (%)	1225 (97.2)	880 (88.4)	
IRCCS CRO			
Total prescriptions	N = 831	N = 222	
ADR			<0.001
Yes (%)	0	74 (33.3)	
No (%)	831 (100.0)	148 (66.7)	

**Table 3:** Frequencies of drug prescriptions with at least one associated ADR and the most frequent undesirable effects for each.

Drug	Drug Prescriptions with at Least One Associated ADR (%) N = 190	Most Frequent Undesirable Effect
Methotrexate	27 (14.2)	Neutropenic fever
Doxorubicin	26 (13.7)	Neutropenic fever
Ifosfamide	24 (12.6)	Pancytopenia
Vincristine	22 (11.6)	Neutropenic fever
Cytarabine	21 (11.1)	Neutropenic fever
Etoposide	20 (10.5)	Pancytopenia, Neutropenic fever
Cyclophosphamide	15 (7.9)	Neutropenic fever
Cisplatin	5 (2.6)	Neutropenia
Crisantaspase	4 (2.1)	Gallstones, Gastroenteritis, Infectious hepatitis, Hyperammonemia, Hyponatremia
Daunorubicin	4 (2.1)	Acute renal insufficiency, Vomiting, Decreased fibrinogen
Mercaptopurine	4 (2.1)	Neutropenic fever
Pegaspargase	4 (2.1)	Infective cholangitis, Painful joints, Hepatic steatosis, Rash
Blinatumomab	2 (1.1)	Fever
Fludarabine	2 (1.1)	Fever
Gemtuzumab ozogamicin	2 (1.1)	Fever, Neutropenic fever, Itching
Mitoxantrone	2 (1.1)	Rash, Neutropenic fever
Bortezomib	1 (0.5)	Headache, Neutropenic fever, Prolonged QT
Busulfan	1 (0.5)	Psychomotor agitation
Carboplatin	1 (0.5)	Neutropenic fever, Sepsis
Methylprednisolone	1 (0.5)	Neutropenic fever
Thioguanine	1 (0.5)	Pancytopenia
Vindesine	1 (0.5)	Muscle pain

**Table 4:** Administered oncology drugs that resulted in one or more ADR due to on-label and off-label prescriptions.

	Therapies (%) N = 1218
ADR	190 (15.6)
Number of ADRs	
1	144 (75.8)
2	39 (20.5)
3	3 (1.6)
4	3 (1.6)
5	1 (0.5)
ADRs due to off-label drugs	9 (4.8)
Implicated drugs	
Gemtuzumab ozogamicin	2 (22.2)
Methotrexate	2 (22.2)
Mitoxantrone	2 (22.2)
Bortezomib	1 (11.1)
Carboplatin	1 (11.1)
Cisplatin	1 (11.1)
ADRs due to on-label drugs	181 (14.9)
Implicated drugs	
Doxorubicin	26 (14.5)
Methotrexate	25 (14.0)
Ifosfamide	24 (13.4)
Vincristine	22 (12.3)
Cytarabine	21 (11.7)
Etoposide	20 (11.0)
Cyclophosphamide	15 (8.3)
Cisplatin	4 (2.2)
Crisantaspase	4 (2.2)
Daunorubicin	4 (2.2)
Mercaptopurine	4 (2.2)
Pegaspargase	4 (2.2)
Blinatumomab	2 (1.1)
Fludarabine	2 (1.1)
Other	4 (2.2)

**Table 5:** Frequencies of ADRs classified according to SOC and total number of patients who manifested ADRs at least once.

**SOC Description**	**ADRs (%)** **N = 130**	**Patients** **N = 32**
Blood and lymphatic system disorders	56 (43.1)	25 (78.1)
Gastrointestinal disorders	20 (15.4)	15 (46.9)
Skin and subcutaneous tissue disorders	10 (7.7)	8 (25.0)
General disorders and administration site conditions	8 (6.2)	7 (21.9)
Respiratory, thoracic and mediastinal disorders	7 (5.4)	4 (12.5)
Investigations	7 (5.4)	5 (15.6)
Nervous system disorders	4 (3.1)	3 (9.4)
Hepatobiliary disorders	4 (3.1)	3 (9.4)
Infections and infestations	3 (2.3)	3 (9.4)
Renal and urinary disorders	3 (2.3)	2 (6.3)
Cardiac disorders	2 (1.5)	2 (6.3)
Musculoskeletal and connective tissue disorders	2 (1.5)	2 (6.3)
Metabolism and nutrition disorders	2 (1.5)	2 (6.3)
Other	2 (1.5)	2 (6.3)
**Blood and Lymphatic System Disorders**	**ADRs (%)** **N = 56**	**Patients (%)** **N = 25**
Neutropenic fever	28 (50.0)	16 (64)
Pancytopenia	9 (16.1)	5 (20)
Neutropenia	6 (10.7)	5 (20)
Anemia	5 (8.9)	5 (20)
Leucopenia	5 (8.9)	3 (12)
Thrombocytopenia	2 (3.6)	2 (8)
Hemoglobin low	1 (1.8)	1 (4)
**Gastrointestinal Disorders**	**ADRs (%)** **N = 20**	**Patients (%)** **N = 15**
Vomiting	7 (35)	7 (46.7)
Mucositis oral	5 (25)	4 (26.7)
Diarrhea	3 (15)	3 (20.0)
Nausea	2 (10)	2 (13.3)
Colitis	1 (5)	1 (6.7)
Esophagitis	1 (5)	1 (6.7)
Gastroenteritis Novovirus	1 (5)	1 (6.7)

**Table 6:** ADR outcome frequencies on the total of ADRs collected and number of patients in whom they happened at least once.

ADR Outcome	ADR (%) N = 130	Patients (%) N = 32
Complete resolution	81 (62.3)	29 (90.6)
Improvement	33 (25.5)	18 (56.3)
Worsening or not improving	6 (4.6)	4 (12.5)
Resolution with consequences	5 (3.8)	3 (9.4)
Not reported	5 (3.8)	5 (15.6)

**Table 7:** Drugs involved in interactions and their frequencies.

Drugs	Therapies (%) N = 266
Methotrexate	45 (16.9)
Sulfamethoxazole/Trimetoprim	37 (13.9)
Mercaptopurine	20 (7.5)
Amoxicillin/Clavulanate	12 (4.5)
Amphotericin B	12 (4.5)
Ciprofloxacin	12 (4.5)
Cyclophosphamide	10 (3.8)
Fluconazole	8 (3.0)
Risperidone	8 (3.0)
Other	102 (38.3)

**Table 8:** Risk rating and type of interaction in ADRs associated with interactions, and in patients with at least one ADR associated with interactions.

	**ADRs Associated with Interactions**		***p*** **-Value**
	**No (%)** **N = 86**	**Yes (%)** **N = 47**	
Risk rating of interaction			0.170
Avoid combination	4 (4.7)	2 (4.3)	
Consider therapy modification	11 (12.8)	12 (25.5)	
Monitor therapy	71 (82.5)	33 (70.2)	
Type of interaction			<0.001
Pharmacokinetics	31 (36.1)	34 (72.3)	
Pharmacodynamics	55 (63.9)	13 (27.7)	
	**Patients with at Least One ADR Associated with Interactions**		
	**No (%)** **N = 7**	**Yes (%)** **N = 24**	
At least one of the following risk ratings of interactions associated with ADR			
Avoid combination	0	2 (8.3)	
Consider therapy modification	3 (42.8)	10 (41.7)	
Monitor therapy	6 (85.7)	21 (87.5)	
At least one of the following types of interactions associated with ADR			
Pharmacokinetics	4 (57.1)	18 (75.0)	
Pharmacodynamics	6 (85.7)	11 (45.8)	

## Data Availability

Data available on request due to restrictions. The data presented in this study are available on request from the corresponding author. The data are not publicly available due to privacy or ethical restrictions.
